# Viral genome packaging terminase cleaves DNA using the canonical RuvC-like two-metal catalysis mechanism

**DOI:** 10.1093/nar/gkw1354

**Published:** 2017-01-18

**Authors:** Rui-Gang Xu, Huw T. Jenkins, Maria Chechik, Elena V. Blagova, Anna Lopatina, Evgeny Klimuk, Leonid Minakhin, Konstantin Severinov, Sandra J. Greive, Alfred A. Antson

**Affiliations:** 1 York Structural Biology Laboratory, Department of Chemistry, University of York, York YO10 5DD, UK; 2 Institute of Molecular Genetics, Russian Academy of Sciences, Moscow 123182, Russia; 3 Skolkovo Institute of Science and Technology, Skolkovo 143025, Russia; 4 Waksman Institute for Microbiology, Rutgers, The State University of New Jersey, NJ 08854, USA

## Abstract

Bacteriophages and large dsDNA viruses encode sophisticated machinery to translocate their DNA into a preformed empty capsid. An essential part of this machine, the large terminase protein, processes viral DNA into constituent units utilizing its nuclease activity. Crystal structures of the large terminase nuclease from the thermophilic bacteriophage G20c show that it is most similar to the RuvC family of the RNase H-like endonucleases. Like RuvC proteins, the nuclease requires either Mn^2+^, Mg^2+^ or Co^2+^ ions for activity, but is inactive with Zn^2+^ and Ca^2+^. High resolution crystal structures of complexes with different metals reveal that in the absence of DNA, only one catalytic metal ion is accommodated in the active site. Binding of the second metal ion may be facilitated by conformational variability, which enables the two catalytic aspartic acids to be brought closer to each other. Structural comparison indicates that in common with the RuvC family, the location of the two catalytic metals differs from other members of the RNase H family. In contrast to a recently proposed mechanism, the available data do not support binding of the two metals at an ultra-short interatomic distance. Thus we postulate that viral terminases cleave DNA by the canonical RuvC-like mechanism.

## INTRODUCTION

The large terminase protein is a key component of the DNA packaging machinery in tailed bacteriophages and evolutionarily related herpes viruses (1,2). Typically, in addition to an ATPase domain which powers DNA translocation (3,4), the large terminase contains a nuclease domain which cuts concatemeric DNA, generated by rolling circle replication (1,5,6). The nuclease cleaves the DNA concatemer first in the initiation phase and later in the completion stage of the DNA packaging process (7). After the first cut, the nascent genome end, in complex with the large terminase motor assembly, is docked onto the portal vertex of the empty procapsid (7) to enable DNA translocation into this protective container. Unlike phage λ where the nuclease cuts at the specific c*osN* site (8), in other phages such as T4, SPP1 and P22, only the first cut is made at a specific sequence close to the packaging *(pac)* site while the second cut is non-sequence specific (9–11). This second, or headful, cleavage event is made after around 102 to 110% of a genome length DNA has been packaged into the procapsid (12).

It has been assumed that the large terminase nuclease utilizes the two-metal catalysis mechanism proposed for other members of the RNase H-like endonucleases (13) such as RNase H, transposases, retroviral integrases and RuvC Holliday junction resolvases. This assumption is supported by two observations: firstly, the large terminase nuclease domain resembles the RNase-H fold (14–19). Secondly, simultaneous binding of two metals, occupying positions A and B, has been observed in crystal structures of human cytomegalovirus (HCMV) UL89 nuclease and in the structure of the Sf6 gp2 nuclease in complex with β-thujaplicinol (14,18). In general, the catalytic mechanism involving two metal ions was previously proposed for phosphoryl transfer reactions catalyzed by DNA polymerase I 3΄, 5΄-exonuclease, alkaline phosphatase, RNase P, group I and group II self-splicing introns and spliceosome (20–22). During the catalysis, the two metal ions form inner-sphere complexes with the scissile phosphate, the active site carboxylates and coordinated water molecules (Supplementary Figure S1). Metal A activates a coordinated water or sugar hydroxyl for nucleophilic attack, while metal B stabilizes the oxyanion leaving group in the transition state (20). More recent studies suggested that metal B is driving the reaction forward via energetically favorable transformation from an irregular dehydrated five-ligand coordination into a hydrated octahedral coordination (23,24).

A structural study on the *Bacillus halodurans* RNase H (Bh-RNase H) complex with an RNA/DNA hybrid suggested that during catalysis, the two metal ions, initially separated by ∼4.0 Å, are likely to move closer together, to ∼3.5 Å distance, neutralizing the developing negative charge of the pentavalent transition state (24) (Supplementary Figure S1). Recently, two different metal binding modes were reported for the Sf6 gp2 nuclease (14). In the first metal binding mode, the two Mg^2+^ or Mn^2+^ ions were modeled at ultra-short metal-metal distances of 2.42 and 2.64 Å, respectively, whereas in the second mode the two Mn^2+^ ions are separated by 3.75 Å. It was argued that binding two metals at the ultra-short metal-metal distance generates a highly positive electrostatic niche, driving the formation of the transition state (14).

Here, we present high resolution structures of the large terminase nuclease domain from *Thermus thermophilus* (Tth) bacteriophage G20c, a close relative of bacteriophages P74-26 and P23-45 (25). Structure comparison reveals plasticity in loop L_1_, which we propose plays an important role in facilitating nuclease activity during interaction with DNA. Structures of nuclease complexes with different divalent metal ions and their comparison with structural information on other members of the RNase H-like endonucleases, along with mutational and nuclease activity data, allow re-examination of the catalytic mechanism. This analysis supports a canonical RuvC-like mechanism for G20c and other viral large terminase nucleases, that does not involve bringing the two metals to an ultra-short distance.

## MATERIALS AND METHODS

### Phage isolation and sequencing

G20c was isolated from a natural hot water source with a temperature of ∼65 °C and pH 7.5 (Geyzer Valley, Kamchatka peninsula) using Tth HB8 strain as a host. Phage infection, isolation of individual plaques, preparation of phage lysate and phage genomic DNA purification and sequencing were performed as described for phages P74-26 and P23-45 (25). The percentage of G20c synteny to P74-26 and P23-45 (by total genome alignment) is 95 and 94%, respectively. Blastn analysis of ORFs of G20c reveals that 105 out of 111 ORFs are highly similar (*e*-value less than 1E-33) to those of P23-45 and/or P74-26.

### Cloning, expression and purification

The DNA fragment encoding either the full length G20c large terminase (residues 1–485) or the nuclease domain (residues 257–443) were amplified by PCR and cloned into the vector pET-YSBLIC3C by using ligation-independent cloning (26). In this vector, the protein coding sequence is joined to a sequence encoding for an N-terminal 6-histidine tag fused to the human rhinovirus 3C protease cleavage site. Site directed mutagenesis was used to introduce codon changes for all the mutants using the CloneAmp™ HiFi PCR Premix (Takara Bio USA, Inc). The full-length terminase and the nuclease domain together with all the mutants were expressed using the same protocol in *E. coli* Rosetta (DE3) pLysS (Novagen EMD Millipore, USA) in LB medium containing 30 μg/ml kanamycin and 34 μg/ml chloramphenicol. Cells were grown at 37°C until OD_600_ reached 0.6–0.8 followed by induction with 1mM isopropyl 1-thio-β-D-galactopyranoside and further growth for 2 h. Cells were harvested by centrifugation for 20 min at 5000 × *g* at 4°C and frozen at −80°C before purification.

Before sonication, cell pellets were resuspended in buffer A (20 mM Tris pH 7.5, 1 M NaCl) containing 1 mM AEBSF, 0.5 μg/ml leupeptin, 0.7 μg/ml pepstatin and 0.1 mg/ml lysozyme. The lysate was clarified by centrifugation at 19 000 × *g* for 1 h and filtration using a 0.45 μm filter. Proteins were first purified by nickel affinity chromatography with a His-Trap column (GE Healthcare) equilibrated with buffer A containing 10 mM imidazole, and eluted with a 10–500 mM imidazole linear gradient in buffer A. The eluted target protein fractions were collected and dialyzed into 20 mM Tris pH 7.5, 250 mM NaCl, 0.5 mM DTT at 4°C overnight. During the dialysis, HRV 3C protease was added to the protein in a 1:50 (w/w) ratio to remove the N-terminal 6-His-tag. Protein samples after digestion were applied to the His-Trap column as before. A concentrated flow through was applied to a Superdex 200 Hiload 16/60 column pre-equilibrated in 20 mM Tris-HCl, pH 7.5, and 250 mM NaCl (buffer B). The final protein samples were concentrated to 20–100 mg/ml.

### Crystallization, data collection, structure determination and refinement

Crystals of the nuclease domain were first obtained from an in-drop proteolysis of the full-length large terminase in 0.1 M MES pH 6.0, 20% (w/v) PEG 6000, 10 mM ZnCl_2_ (Table 1, Crystal form 1). However, these crystals were difficult to reproduce. A nuclease domain construct containing residues 257–443 was then cloned, expressed and purified for crystallization. Before crystallization, the protein was diluted to 10 mg/ml using 20 mM Tris pH 7.5, 50 mM NaCl solution. Crystallization was performed at 20°C using sitting drop vapor diffusion by mixing 0.5 μl of the protein solution with 0.5 μl of reservoir solution, before equilibrating against 100 μl of the reservoir solution. Crystals for form 2 (Table 1), space group P3_2_21, grew from 0.2 M lithium sulphate, 0.1 M Bis-Tris pH 5.5, 25% (w/v) PEG 3350. Crystals were soaked in a cryo-protectant solution containing 0.2 M lithium sulphate, 0.1 M Bis-Tris pH 5.5, 30% (w/v) PEG 3350 and 1 mM CoCl_2_ for 20 seconds before vitrification in liquid nitrogen. As Co^2+^ was not observed in the electron density, we refer to this form as "Apo". Crystal form 3 (Table 1), space group P2_1_, was obtained using 0.2 M ammonium tartrate, 0.1 M Bis-Tris pH 5.5, 20% (w/v) PEG 3350. To produce crystals with bound divalent metal ions, crystals belonging to crystal form 3 were soaked in a cryo-protectant solution containing 0.2 M ammonium sulphate, 0.1 M Bis-Tris pH 5.5, 30% (w/v) PEG 3350 with 50 mM MnCl_2_ / CaCl_2_ or 10 mM MgCl_2_/CoCl_2_ for 3 min before flash cooling in liquid nitrogen.

**Table 1.** X-ray data collection and refinement statistics

Crystal form	1 Zn^2+^-SAD	1 Zn^2+^	2 Apo	3 Mg^2+^	3 Mn^2+^	3 Co^2+^	3 Ca^2+^
Wavelength (Å)	1.2841	0.9763	0.9795	0.9796	0.9795	0.9796	0.9795
Space group	P6_5_	P6_5_	P3_2_21	P2_1_	P2_1_	P2_1_	P2_1_
Unit-cell a, c (Å)	60.8, 90.4	60.9, 90.4	55.1, 95.7	43.8, 69.9	43.8, 70.0	42.9, 69.7	43.4, 69.8
Unit-cell b (Å)				54.0	53.9	53.9	53.6
Unit-cell β (°)				91.9	92.1	91.6	91.8
Resolution (Å)	45.53-1.60 (1.63-1.60)	45.60-1.45 (1.47-1.45)	47.72-2.15 (2.21-2.15)	43.78-1.20 (1.22-1.20)	43.78-1.20 (1.22-1.20)	42.92-1.60 (1.63-1.60)	43.43-1.10 (1.11-1.10)
R_merge_ (%)	7.9 (147.6)	8.6 (129.1)	4.3 (80.4)	7.3 (76.5)	4.2 (34.6)	7.2 (71.9)	7.8 (97.7)
I/σ<I>	13.4 (1.7)	8.2 (1.0)	10.3 (1.0)	7.4 (1.6)	17.6 (4.0)	6.7 (1.1)	6.0 (1.0)
Completeness (%)	100.0(100.0)	99.7(99.5)	95.6 (90.6)	97.0 (97.6)	95.0 (89.1)	95.5 (84.6)	98.8 (87.3)
Multiplicity	8.8 (8.8)	4.5 (3.9)	2.2 (2.2)	2.5 (2.5)	4.3 (4.3)	2.1 (2.0)	3.2 (2.8)
CC_1/2_	0.998 (0.746)	0.997 (0.420)	0.999 (0.365)	0.996 (0.522)	0.999 (0.906)	0.997 (0.611)	0.996 (0.385)
Refinement							
R_work_/R_free_		0.201/0.211	0.200/0.280	0.183/0.209	0.169/0.182	0.185/0.224	0.222/0.235
Mean B factor (A^2^)		23	56	14	11	22	12
R.M.S.D.							
Bond lengths (Å)		0.008	0.009	0.007	0.006	0.007	0.006
Bond angles (°)		1.2	1.2	1.2	1.2	1.2	1.2
Ramachandran (%)							
Favored		98	97	99	98	99	98
Allowed		2	3	1	2	1	2
Outliers		0	0	0	0	0	0

Values in the parentheses are for the outermost resolution shell.

SAD, single-wavelength anomalous dispersion.

Diffraction data were collected at Diamond Light Source beamlines I02, I03 and I04 (Table 1) and processed using XDS (27). The structure of the crystal form 1, containing bound Zn^2+^, was determined by single-wavelength anomalous dispersion (SAD) using SHELXD (28). Density modification was performed by SHELXE (29), followed by model building by ARP/wARP (30). Structures of the apo form and metal complexes were determined by molecular replacement, using Phaser (31). Refinement was carried out using REFMAC5 (32), accompanied by iterative model building with Coot (33). Chimera (34) and CCP4mg (35) were used for figure generation.

### Examination of Sf6 structure

The mFo-DFc maps for the Sf6 gp2 nuclease with two modeled Mg^2+^ ions (PDB code: 5C12) or Mn^2+^ ions (PDB code: 5C15) were generated using phenix.maps by omitting the two modeled metal ions and surrounding water molecules in the active site (water molecules 908, 910, 972, 995 and 1027 for the 5C12 structure and water molecules 999, 976, 923 and 1096 for the 5C15 structure). To avoid any differences resulting from software versions, we used phenix.maps from the same (1.8.1_1168) version of Phenix (36) as phenix.refine (37) used by Zhao *et al*. (14).

### Metal ion removal

Residual metal ion contaminants co-purified with either the protein samples or DNA substrate were removed using Chelex^®^ 100 resin (Bio-Rad Laboratories, Inc.). Approximately 50 μl of the resin slurry was used for a 100 μl protein sample. The beads were first dried by filter centrifugation and the pellet then added directly into the protein sample. This was left to shake gently for 1 h before the protein was collected using a 0.22 μm benchtop Corning^®^ Costar^®^ Spin-X^®^ centrifuge tube filter (Sigma-Aldrich, Inc.)

### *In vitro* nuclease assays

The G20c nuclease is active in the temperature range 20–60°C. 37°C was chosen for incubation, as at this temperature the nuclease fully digested the DNA substrate in 20 min (Supplementary Figure S2). A total of 120 ng of supercoiled or EcoRI-linearized pUC18 DNA containing the SPP1 pacL site were used as generic DNA substrates, and incubated with the purified G20c large terminase protein (1 μM) in a 20 μl reaction mixture containing 7 mM HEPES pH 7.5, 7 mM potassium glutamate with various concentrations of divalent metal ions at 37°C for 30 min, unless otherwise stated. The reaction was stopped by the addition of EDTA (50 mM), SDS (0.5%) and proteinase K (50 μg/ml) with a further incubation at 37°C for 30 min. The resultant cleavage products were then separated on a 0.8 or 1.0% agarose gel (1 × TAE running buffer) followed by ethidium bromide staining.

## RESULTS

### Structure of the G20c large terminase nuclease

**Figure 1.**
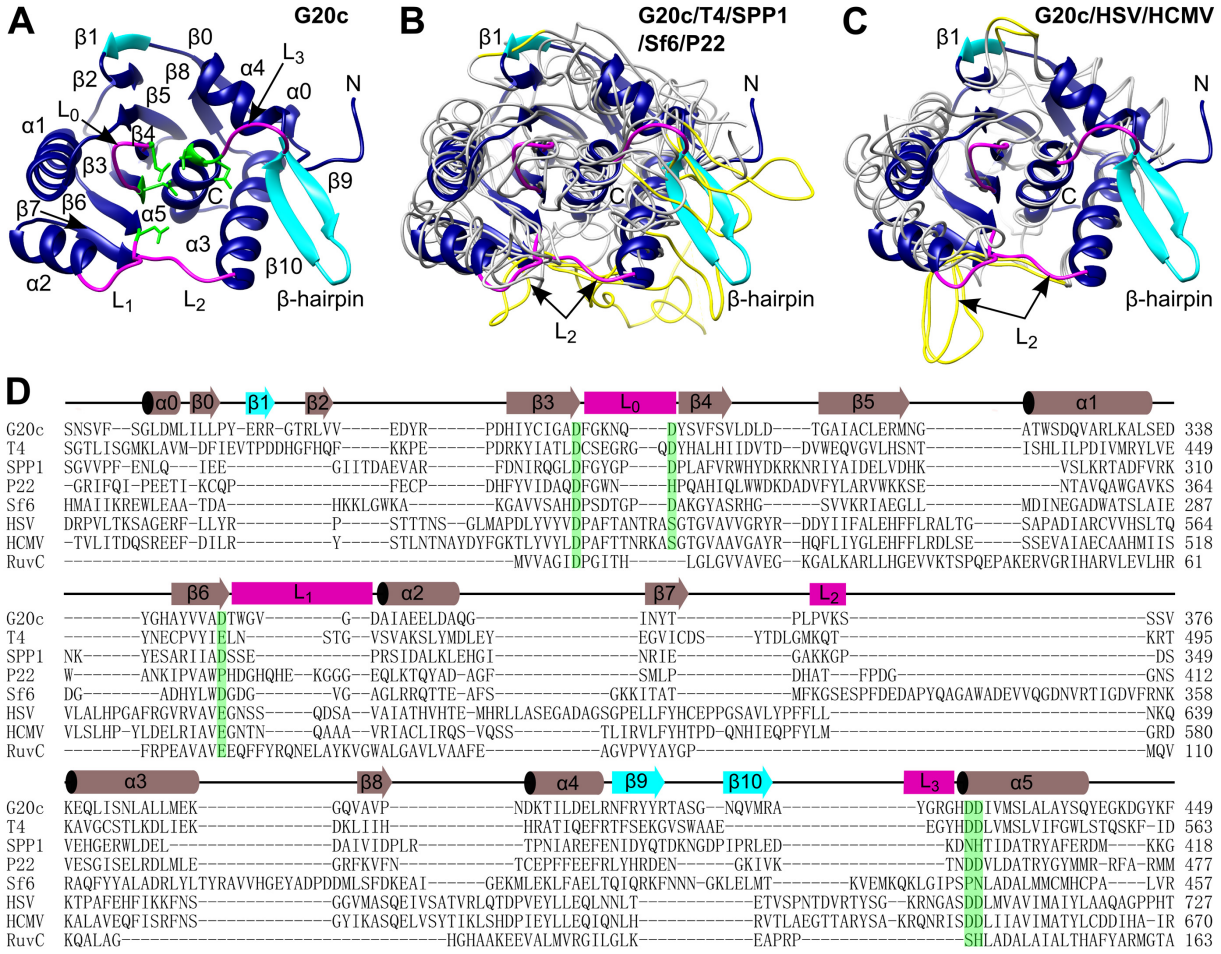
Comparison of large terminase nuclease domains from different viruses. (**A**) Ribbon diagram of the G20c large terminase nuclease. β1 and β-hairpin (β9 and β10) are highlighted in cyan. Loop L_0_, L_1_, L_2_ and L_3_ are colored in magenta. Active site carboxylates are shown in green. (**B**) Superposition of the G20c nuclease (blue/cyan ribbon, as in panel A) with large terminase nucleases from T4, SPP1, Sf6 and P22 (gray chain traces). Segments equivalent to L_2_, β1 and the β-hairpin of the G20c nuclease are in yellow. (**C**) The same superposition as in (B) but with the herpesviruses HSV and HCMV nucleases. (**D**) Structure based sequence alignment of large terminase nucleases from bacteriophages G20c, T4, SPP1, P22, Sf6; herpesviruses HSV, HCMV and *Thermus thermophilus* RuvC resolvase. Secondary structure elements of the G20c nuclease are shown above the alignment. Conserved/equivalent active site residues are highlighted in green.

Initial crystals were obtained from a proteolytically cleaved C-terminal fragment of the full-length protein (Crystal form 1, Table 1). A bound Zn^2+^ ion from the crystallization solution enabled the structure to be determined by SAD. Subsequently a recombinant protein construct, residues 257 to 443, corresponding to the nuclease domain, crystallized in two different crystal forms, 2 and 3 (Table 1). Crystal forms 1 and 2 contain a single molecule, whereas crystal form 3 contains two protein molecules per asymmetric unit. The overall structure adopts the RNase-H fold (Figure 1A). As in other members of the RNase H-like endonucleases, a cluster of carboxylic acids is contributed to the active site by strands β3, β4 and β6, helix α5 and loops L_0_–L_3_. These residues were shown to be critical for bacteriophage function, DNA packaging or nuclease activities in bacteriophages T4 (17,38) and SPP1 (16,39). Loops L_1_ and L_2_, defined earlier for the P22 large terminase nuclease (15), correspond to residues 347–352 and 369–372, respectively, in the G20c large terminase. The two other loops, L_0_ and L_3_, residues 295–301 and 423–427, respectively, also contribute to the active site. The β-hairpin (β9 and β10 strands on Figure 1A), a unique feature of viral large terminases not observed in other members of the RNase H-like endonucleases (16), is well ordered in crystal forms 2 and 3, but is invisible in the structure of the proteolytic fragment (crystal form 1).

### Comparison with other viral nucleases

Superposition of the G20c large terminase nuclease with bacteriophage (14–16,40) and herpes virus (18,19) nucleases reveals highly similar three-dimensional structures (Figure 1B and C), despite low sequence identity (Figure 1D). The highest similarity with phage nucleases is for T4 gp17 (40) (C_α_ rmsd of 1.9 Å for 176 residues that exhibit 25.4% sequence identity), while the lowest similarity is with the SPP1 G2P (16) (C_α_ rmsd of 2.9 Å for 143 residues with 12.6% sequence identity). C_α_ rmsd with the HSV pUL15 (19) (135 residues, 20.5% sequence identity) and HCMV UL89 (18) (141 residues, 19.2% sequence identity) nucleases is 2.4 and 2.5 Å, respectively.

The G20c nuclease has three major structural differences compared with nucleases from other viruses. Firstly, an additional β-strand (β1 on Figure 1A) extends the central β-sheet. Secondly, the uniquely viral terminase β-hairpin (strands β9 and β10 in Figure 1A), is more extended and better ordered (Figure 1B). Thirdly, loop L_2_ (‘hairpin’ in (19)) implicated in interaction with DNA in the HSV pUL15 nuclease is much shorter (Figure 1C).

### Metal dependence of nuclease activity

**Figure 2.**
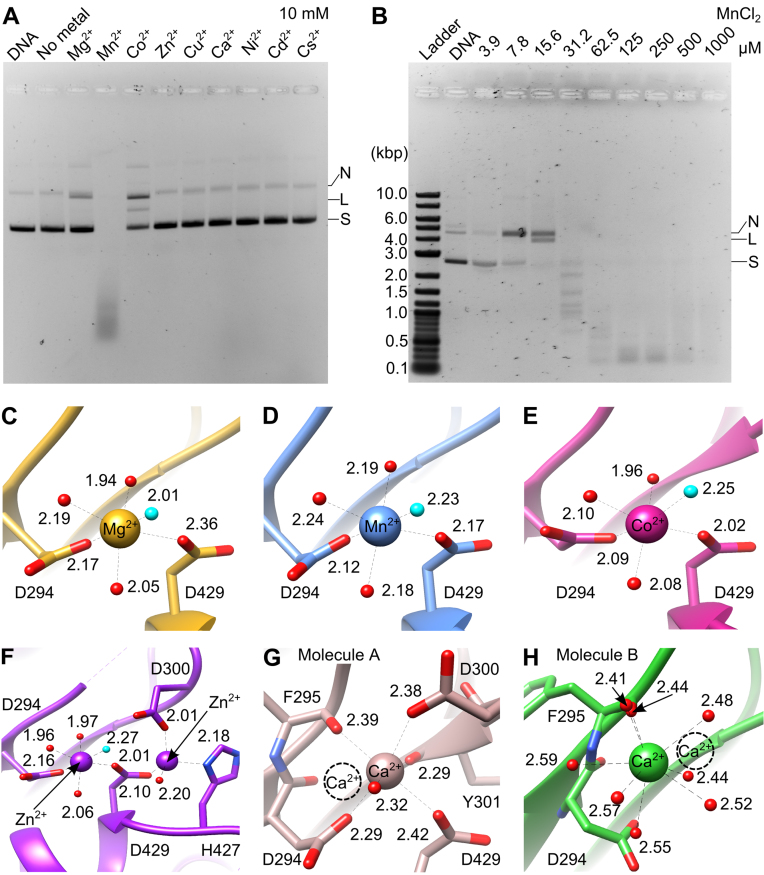
Dependence of nuclease activity on metals and metal coordination in crystal structures. (**A**) Effect of divalent metal ions on the nuclease activity. N, nicked; L, linear; S, supercoiled DNA. (**B**) Effect of MnCl_2_ concentration on the nuclease activity: Ladder: 2-log DNA ladder (New England Biolabs). Metal coordination in crystal structures of the G20c nuclease in complex with (**C**) Mg^2+^, (**D**) Mn^2+^, (**E**) Co^2+^, (**F**) Zn^2+^, (**G** and **H**) Ca^2+^. The Ca^2+^ ion from the second molecule in the asymmetric unit is shown as dashed circle. The nucleophilic water molecule is highlighted in cyan.

In order to easily detect the catalytic activity, assays were performed in low salt conditions, to facilitate binding of DNA and divalent metal ions. Similar to T4 and other headful phages, the nuclease activity appears to be non-sequence specific under these conditions. The nuclease was active in the presence of Mn^2+^, Mg^2+^ and Co^2+^ but inactive with Ni^2+^, Zn^2+^ or Ca^2+^ (Figure 2A and Supplementary Figure S2), consistent with observations for T4 gp17 (41), SPP1 G2P (39) and HCMV UL89 (18). Addition of Cu^2+^, Cd^2+^ and Cs^2+^ also did not support catalysis. Similar to G2P and UL89, addition of Mg^2+^ resulted only in limited activity (18,39), leading to production of nicked or linearized DNA when supercoiled DNA was used as substrate. However, G20c nuclease had minimal activity with Co^2+^, in contrast to the high non-specific *in vitro* nuclease activity observed for SPP1 G2P nuclease (comparable with Mn^2+^ (39)) or the absence of activity for the T4 gp17 nuclease (41). Significantly, in our assay conditions, Mn^2+^ supported the nuclease activity of the G20c large terminase even at very low (μM) concentrations, producing DNA segments with defined length (Figure 2B), suggesting some sequence preference for cleavage.

### Structures of nuclease-metal complexes

Structures for complexes with Mn^2+^, Mg^2+^, Co^2+^ and Ca^2+^ were determined by soaking crystals of the apo protein, whereas the structure of the Zn^2+^ complex was obtained by co-crystallization (Supplementary Figure S3). Only one metal ion, bound to the site A, was identified for conditions containing Mg^2+^, Mn^2+^ and Co^2+^. This metal ion is coordinated by the side chains of D294 and D429 and four water molecules in the canonical octahedral geometry (42) (Figure 2C–E). A similar coordination is observed for Zn^2+^, although there is also a second Zn^2+^ ion bound at an additional satellite site, at a distance of 4.6 Å from the site A Zn^2+^ ion (Figure 2F). Finally, two different Ca^2+^ binding modes are observed in each of the two protein molecules present in the asymmetric unit (Figure 2G and H).

**Figure 3.**
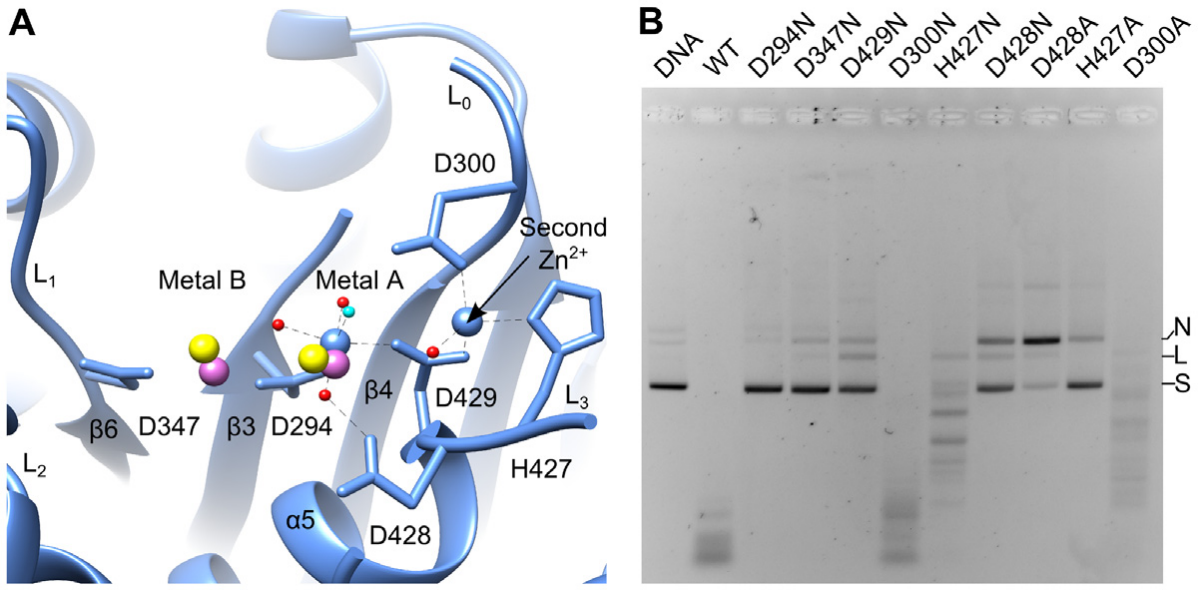
Metal binding sites. (**A**) Active site of the G20c nuclease complex with Zn^2+^. The two Mn^2+^ ions, taken from structure superposition with SPP1 and HCMV nuclease-Mn^2+^ complexes are in magenta and yellow, respectively. The water nucleophile is shown in cyan. (**B**) *In vitro* nuclease assays. Activity is shown for the wild-type and active site mutant proteins. N, nicked; L, linear; S, supercoiled DNA.

Superposition of the G20c nuclease structure containing two Zn^2+^ ions (Crystal form 1, Table 1) with SPP1 G2P and HCMV UL89 nucleases containing two bound Mn^2+^ ions (16,18) shows that while one Zn^2+^ ion is bound at site A, the second Zn^2+^ ion is bound on the opposite side of site B (Figure 3A). This Zn^2+^ ion is in a tetrahedral coordination (42) with D429, H427, D300 and a solvent molecule.

### Effect of active site residues on nuclease activity

The functional importance of the metal binding sites A, B and the satellite Zn^2+^ binding site was investigated using full-length protein containing both ATPase and nuclease domains. Aspartic acids coordinated by metals A and B were replaced by asparagine: D294N, D429N, D347N. Nuclease assays for all mutant proteins were performed *in vitro* in the presence of 0.1 or 1 mM MnCl_2_ (Figure 3B and Supplementary Figure S4). Wild-type large terminase converted the entire supercoiled DNA substrate into a smear of shorter DNA fragments at 1 mM MnCl_2_, while at 0.1 mM longer fragments with a somewhat defined length were observed. The lowest nuclease activity was observed for the D294N, D347N and D429N mutant proteins at both concentrations of MnCl_2_ (Figure 3B and Supplementary Figure S4). In contrast, D300N showed nuclease activity comparable to that of the wild-type protein whereas a modest decrease in nuclease activity was observed for D300A. A reduction of the nuclease activity was also observed for the H427N mutation. However, this mutant protein retained the ability to process longer DNA into smaller fragments, even at low (0.1 mM) MnCl_2_ concentrations (Figure 3B). Replacing this residue with alanine (H427A) resulted in deficiency in digestion of the supercoiled DNA at 0.1 mM MnCl_2_ concentration, like for the D347N mutation. This activity was partially recovered at 1 mM MnCl_2_ concentration where the H427A mutant protein could convert the entire supercoiled DNA into nicked and linearized DNA (Supplementary Figure S4). Both the D428N and D428A mutant proteins showed a significant drop in nuclease activity and were deficient for production of shorter DNA fragments (Figure 3B). These mutant proteins, like H427A, exhibited a slight increase in nuclease activity at 1 mM MnCl_2_ (Supplementary Figure S4).

## DISCUSSION

### Structural features

The structure of the nuclease from the thermophilic G20c bacteriophage is very similar to P74-26 large terminase (43), but differs from its mesophilic counterparts by shortened surface loops, notably a shorter loop L_2_ than that present in other bacteriophage and herpes virus nucleases (Figure 1B and C). There is also an increased number of salt bridges (44): 9 versus 5 to 7 found in nucleases of mesophilic viruses (14–16,18,19,40). In addition, the β-hairpin, present only in viral nucleases, is more extended and ordered (Figure 1B). These differences are expected to increase the protein stability at the higher environmental temperatures encountered by the G20c bacteriophage (45).

### Role of active site residues

Mutational analysis indicated that metal coordinating residues D294, D347 and D429 are indispensable for catalysis (Figure 3B). Similar observations were made for SPP1 G2P nuclease (16,39) and for other members (46–48) of the RNase H-like nucleases. Taken together, the data support the two-metal catalysis mechanism, proposed earlier by Nowotny and Yang for the RNase H-like proteins (13,49).

Of the residues that co-ordinate the second Zn^2+^ ion, D300 is conserved among the large terminases of bacteriophages T4, RB49, SPP1 and Sf6, with the equivalent residue in T4 gp17 (D409) reported to be crucial for bacteriophage function (38). Comparison of the catalytic activity for mutants D300N and D300A, indicates that the negative charge of D300 is not essential for catalysis (Figure 3B and Supplementary Figure S4). The slight reduction in activity observed for D300A may be caused by disturbance of the hydrogen bonding network affecting D429 and/or bound DNA.

Histidine and glutamic acid residues adjacent to the metal A site in RNase H proteins have been suggested to play important roles in catalysis by affecting product release (50,51) and/or binding to a third Mg^2+^ during catalysis (52). This can explain the significant reduction in nuclease activity observed for the H427A mutant (Figure 3B and Supplementary Figure S4). Intriguingly, an equivalent serine residue found in the Sf6 gp2 nuclease forms a hydrogen bond with an oxygen atom on the bound metal chelator, occupying the position where the water nucleophile is normally coordinated by metal A (Supplementary Figure S5). Likewise, H427 may be involved in orienting the water nucleophile during the catalysis. This can be facilitated by the conformational flexibility of loop L_3_ and the β-hairpin.

The catalytic deficiency of D428 mutants (Figure 3B and Supplementary Figure S4) suggests that this residue may be responsible for stabilization of metal A binding, since it is proximal to metal A and forms a hydrogen bond with a coordinating inner shell water molecule (Figure 3A).

### Structural basis for metal dependence of nuclease activity

RNase H-like endonucleases require divalent metal ions such as Mg^2+^ or Mn^2+^ for catalysis (46,53–55). The lack of activity in the presence of Ca^2+^ can be explained by the different coordination observed for this ion (Figure 2G and H), induced by its larger atomic radius and longer coordination distances, as compared to Mg^2+^ or Mn^2+^. A similar effect was observed for other RNase H-like enzymes in the presence of Ca^2+^ (50,56).

Due to the similarity in atomic radius, it was suggested that Zn^2+^ can substitute Mg^2+^ in catalysis (23). The activity was shown to be abrogated (56–58) or significantly reduced (51,59) by Zn^2+^ for the RNase H-like endonucleases. However, the structural basis for the reduction in activity remained unclear. In our structure, complexed with two Zn^2+^ ions, the Zn^2+^ ion bound at catalytic site A adopts an octahedral geometry (Figures 2F and 3A), resembling the canonical coordination of Mg^2+^ (Figure 2C) that would support catalysis. However, the second Zn^2+^ bound at an adjacent binding site, not previously reported for the RNase H-like endonucleases, is coordinated by catalytically important residues D429 and H427 (Figures 2F and 3A). Binding of this second Zn^2+^ ion perturbs charge distribution in the active site and may affect DNA and metal B binding as well as water nucleophile formation and coordination.

### Re-examination of metal binding in Sf6 large terminase nuclease

**Figure 4.**
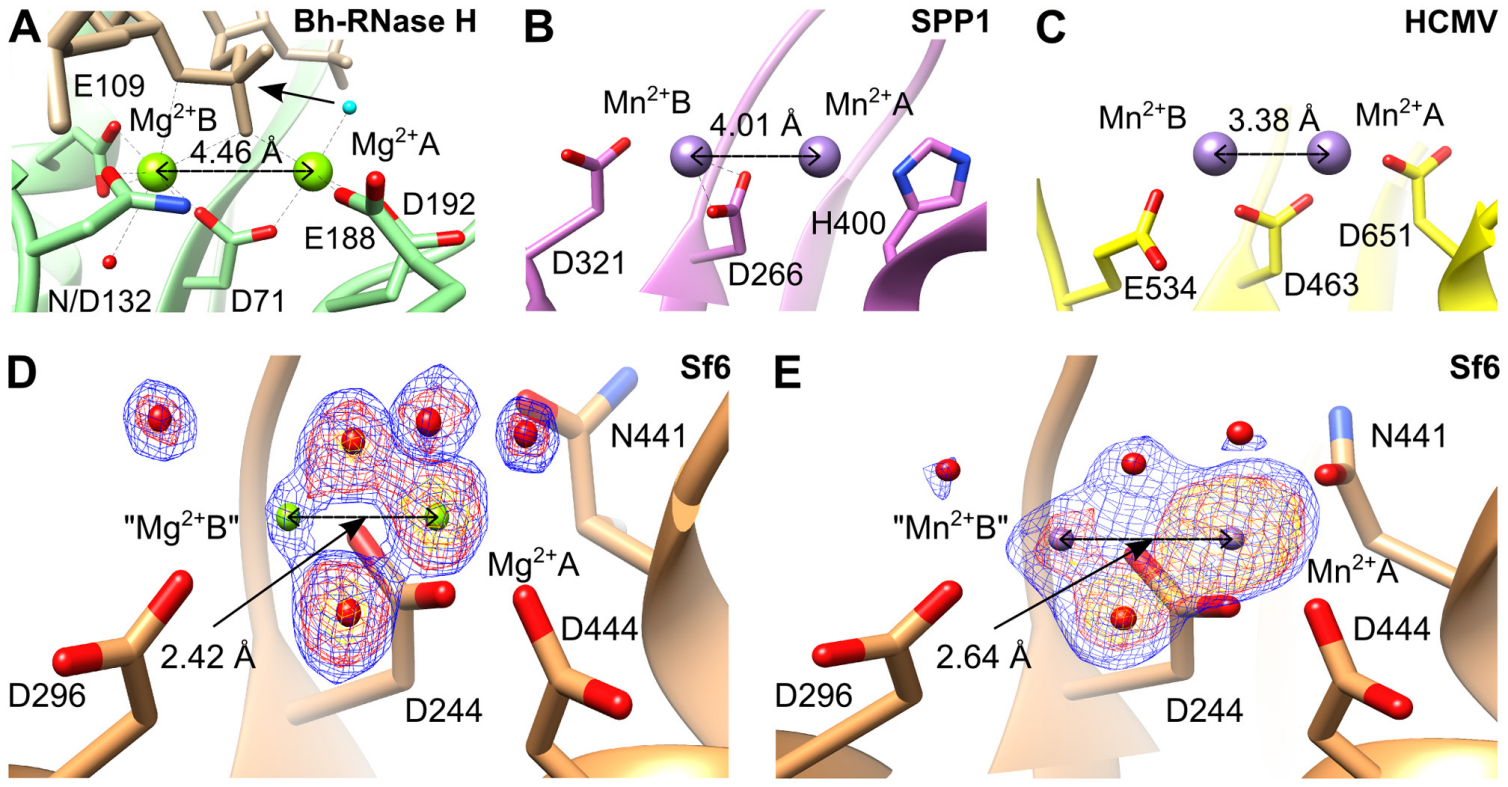
Metal–metal distances observed in crystal structures. (**A**) Active site of *Bacillus halodurans* RNase H complex with RNA/DNA hybrid and Mg^2+^ ions. The RNA is in beige and the nucleophile water molecule is in cyan. (**B–E**) Large terminase nucleases from (B) SPP1, (C) HCMV, (D) Sf6 with bound Mg^2+^ and (E) Sf6 with bound Mn^2+^. Models in panels (D) and (E) are overlaid with mFo-DFc electron density maps calculated after omitting metals and coordinated water molecules, contoured at 12 σ (yellow), 8 σ (red) and 6 σ (blue) in panel (D) and at 12 σ (yellow), 10 σ (red) and 6 σ (blue) in panel (E).

Structural observations for RNase H-like nucleases utilising the two-metal catalysis mechanism, show that the two metal ions, in the presence of the scissile phosphate, jointly coordinated by a conserved aspartic acid, are separated by 3.4–4.5 Å (13,24,50,60) (Figure 4A). Additionally, in the absence of bound DNA substrate the two manganese ions are separated by 4.0 Å (Figure 4B) and 3.4–3.6 Å (Figure 4C), respectively in SPP1 (16) and HCMV (18) large terminase nucleases. Comparable distances were observed for other enzymes catalysing phosphoryl-transfer by the two-metal catalysis mechanism (61).

However for the Sf6 large terminase, in the absence of the scissile phosphate, the two metal ions were modeled at unusually ultra-short distances of 2.42 Å (Mg^2+^-Mg^2+^) (Figure 4D) and 2.64 Å (Mn^2+^-Mn^2+^) (14) (Figure 4E). We observed that in difference maps generated after omitting the two modeled metal ions and coordinating water molecules, the electron density for the metal at site A is clear whereas only ambiguous density, weaker than that for the coordinating solvent molecules, was observed at site B (Figure 4D and E). In both structures, the refined B-factor of the metal modeled at site B is around twice that of the metal at site A and the coordinating atoms, further indicating inconsistencies with experimental data. Therefore, we suggest that the observed weak electron density at the modeled metal site B presumably results from a low occupancy alternative metal binding position, as observed for another DNA processing protein (62), rather than the presence of two metals at the same time at an ultra-short distance which has not been observed before (13,61). These observations indicate that Sf6 nuclease uses a classical two-metal dependent catalysis mechanism, as described originally for RNase H (24,63) and below for G20c nuclease.

### RuvC is the closest structural homologue of large terminase nuclease

**Figure 5.**
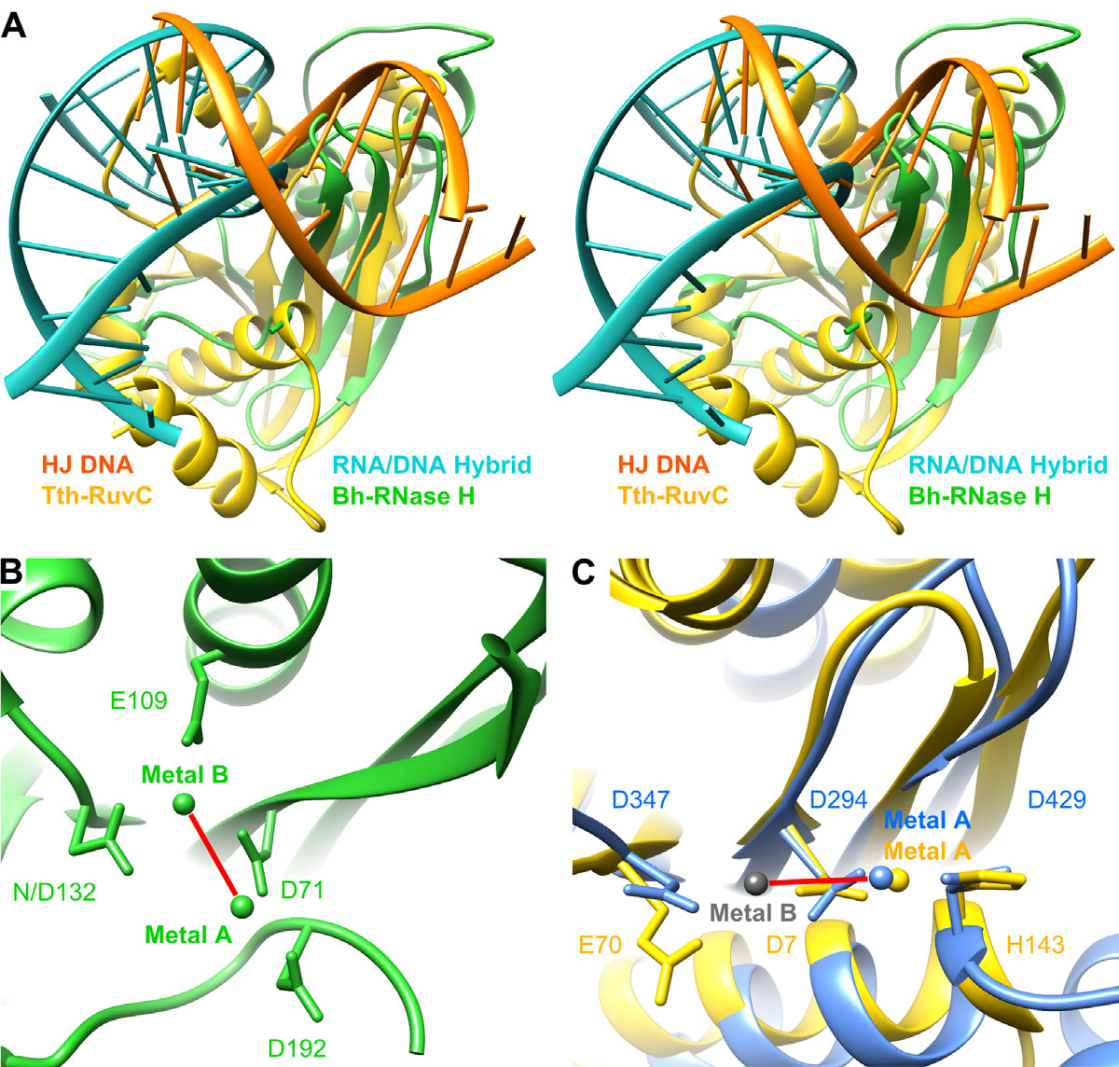
Comparison of metal location and DNA orientation. (**A**) Stereo view showing the superposition of nucleic acid complexes of *Bacillus halodurans* RNase H (green; RNA/DNA hybrid, cyan; PDB accession code 1ZBI) and *Thermus thermophilus* RuvC (yellow; Holliday junction DNA, orange; 4LD0). (**B**) Active site of Bh-RNase H nuclease with bound metal ions. (**C**) Close-up view at the active site in superposed G20c (blue, 5M1N) and Tth-RuvC (yellow, 4EP4) nucleases shown in the same orientation as (B). Bound metal ions (in both structures only at site A) are in corresponding colors. The gray sphere indicates the site B position (as in *Lactococcus* phage bIL67 RuvC, 4KTZ). Note the different relative orientation of the two metals in (B) and (C), indicated by red line.

A DALI search (64) identified RuvC resolvases as the closest structural homologs of the G20c nuclease. Subsequent pairwise secondary-structure matching (SSM) analysis (65) using PDBeFold showed significantly higher *Z*-scores for Tth-RuvC (*Z* = 7.0 for 106 aligned residues) compared with the Bh-RNase H (*Z* = 1.9 for 93 aligned residues), Supplementary Table S1. Interestingly, structural comparison of the Bh-RNase H and Tth-RuvC with bound RNA/DNA hybrid or dsDNA respectively, reveals significant differences (Figure 5A). Notably, a different position of the metal A coordinating residue, D192 in Bh-RNase H versus H143 in Tth-RuvC, was observed (Figure 5B and C). These differences are due to different conformations, i.e. replacement of the extend strand (Bh-RNase H) by an α-helix (Tth-RuvC) which runs in the opposite direction (49,66). Moreover, the additional catalytic residue, E109, coordinated to metal B, is absent in RuvC (46,49,67). These differences result in distinctly different orientations of the active site metals and the bound nucleic acid duplex. It appears that the RuvC family evolved to adjust the position of their metal coordinating residues (and hence metal binding sites) to adapt to different nucleic acid substrates, while maintaining the classic RNase H fold. Structural superposition of the G20c large terminase nuclease with Tth-RuvC, unlike for Bh-RNase H, results in good alignment of the three catalytically important residues (Figure 5C), indicating that RuvC and viral large terminase nucleases utilize a highly similar catalytic mechanism.

### DNA binding surface plasticity

**Figure 6.**
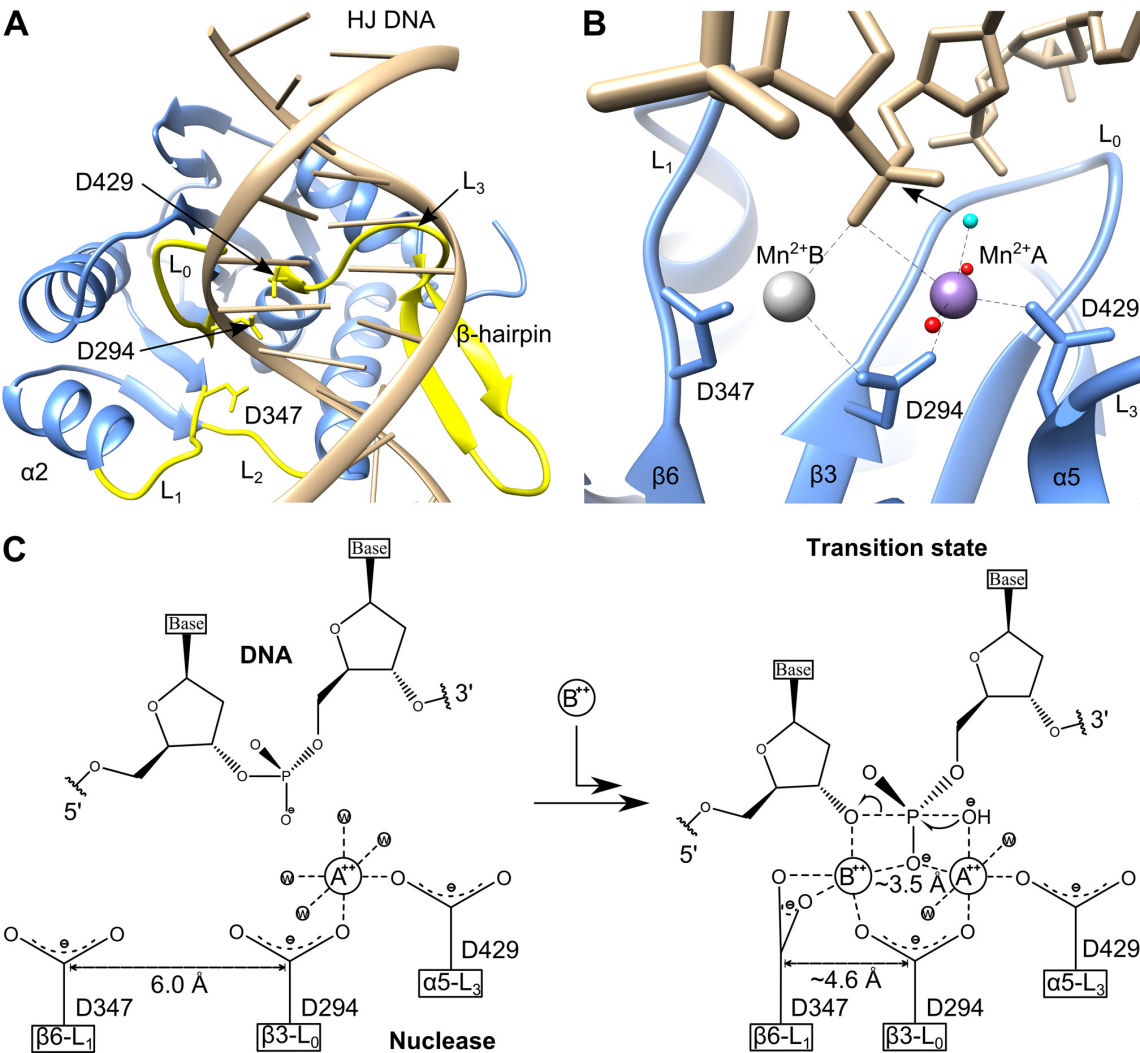
Mechanism of DNA cleavage. (**A**) Model of G20c nuclease complex with DNA. The DNA model was obtained from nuclease superposition with the structure of *Thermus thermophilus* RuvC-DNA complex. Structural segments that are in proximity to the modeled DNA are in yellow. (**B**) Close-up view of the active site of the model (shown in panel A), with Mn^2+^ position at site B (gray sphere) corresponding to its observed position in the crystal structure of SPP1 nuclease. The nucleophile water molecule which is expected to perform the nucleophilic attack on the scissile phosphate is colored in cyan. (**C**) Schematic of the catalytic mechanism showing the proposed movement of D347 toward D294, concomitant with metal B and DNA binding, leading to transition state formation.

Superposition of crystal forms 1, 2 and 3 (Table 1) shows that four loops (L_0_, L_1_, L_2_, L_3_) surrounding the catalytic site and the β-hairpin are flexible and adopt different conformations (Supplementary Figure S6). Importantly, all of these flexible structural segments are conserved both in phage (14–16,40) and herpesvirus (18,19) terminases. We note that D347, implicated in facilitating metal binding at site B during the catalysis (see below), is located at the N-terminus of L_1_. The position of this residue differs significantly between crystal forms 2 and 3 (Supplementary Figure S6), bringing the carboxyl group of D347 1.4 Å closer to the catalytic D294, which is expected to coordinate to both site A and B metals. We also note that a shorter distance between these two residues was observed earlier in two metal bound complexes of SPP1 (16) and HCMV (18) nucleases, and in the *Lactococcus* phage bIL67 RuvC complex with Mg^2+^ (68) (Supplementary Figure S6). A model of the DNA bound to the G20c nuclease was generated by superposition with the structure of the Tth–Ruvc complex with DNA (69) (Figure 6A). In the model, conserved loops L_0_, L_1_, L_2_, that form direct contacts with DNA in the Tth–RuvC resolvase, are in proximity to the DNA (Figure 6A and Supplementary Figure S7). Additionally, Loop L_3_ and the β-hairpin, absent in the Tth–RuvC resolvase, are also in close contact with the modeled DNA, indicating their potential involvement in DNA binding. This is consistent with previous suggestions for the involvement of β-hairpin in interaction with DNA (14–16). Furthermore, the DNA binding region predicted by the modeled G20c–DNA complex presented here is supported by the mutagenesis data for the P74-26 nuclease, reported in the accompanying paper (43).

### RuvC-like, canonical two-metal dependent catalysis

In the model of the G20c nuclease–DNA complex, the site B Mn^2+^ position (Figure 6B) corresponds to the position of the equivalent metal B in the crystal structure of SPP1 G2P (16), with the scissile phosphate of the Holliday junction DNA placed between the two metals. The water nucleophile coordinated by Mn^2+^ A (cyan in Figure 6B) is in proximity to the scissile phosphate, in a position favorable for nucleophilic attack (70).

It has been suggested that in addition to stabilizing the 3΄-leaving group, metal B serves to reduce to the energy barrier between the substrate/product states (23,24). This is facilitated by transformation from the fully dehydrated and irregular coordination in the substrate-bound complex, involving five ligands, into a hydrated octahedral geometry adopted after DNA cleavage. Unlike RNase H family proteins in which the metal site B is surrounded by three carboxylate side chains (Figures 4A and 5B; Supplementary Figure S1), only two conserved carboxylates are present in the large terminase nucleases of bacteriophages T4, SPP1, Sf6, G20c and herpes viruses HCMV and HSV (Figures 4B and C, and 6B; Supplementary Figure S5). Therefore, we suggest that D347 would coordinate metal B in a bidentate conformation (Figure 6C), to allow formation of a similarly dehydrated and irregular coordination at metal B (23,24). Binding of metal B can be facilitated by flexibility in the position of D347, observed in the crystal structures presented here, allowing D347 to move closer to D294. In accordance, structure superposition of the large terminase nucleases from G20c, SPP1, HCMV and other RuvC proteins, show that in the absence of metal B the two aspartate residues are more distant than in its presence (Supplementary Figure S6).

In summary, the following nuclease mechanism can be proposed for viral large terminases. In the absence of DNA, site A is occupied by a divalent metal ion, as in structures of Canarypox virus (71) and Tth*–*RuvC resolvases (72). Upon DNA binding, the negative charge provided by the scissile phosphate facilitates the recruitment of the second metal ion which binds at site B (Figure 6C). Binding of this metal is accompanied by change in the conformation of loop L_1_, bringing D347 closer to metal B, leading to formation of the transition state.

### Insights into headful DNA packaging

The nuclease activity of the large terminase needs to be coupled to- and regulated by- DNA packaging for efficient production of infectious virions. This idea is supported by observations that ATP analogs stimulate the nuclease activity of T4 and P74-26 terminases (41,43). However, recent evidence suggests that this may be indirectly mediated through increased affinity of the ATPase domain of the full-length terminase towards DNA, thereby increasing nuclease activity (3,43). This explanation is consistent with observations that an isolated C-terminal nuclease domain is not as active as full-length large terminase (16) or even completely inactive (15), as observed for SPP1 and P22, respectively.

However, local DNA conformation is also likely to be essential for catalysis, given the similarity with RuvC, which binds branched and distorted DNA. During initiation of DNA packaging, when bacteriophage DNA is recognized by the small terminase protein, the DNA is expected to adopt a bent conformation, which may favor its binding within the active site leading to DNA cleavage (73,74). Finally, when the capsid is filled with DNA, the counteracting forces of the internal pressure of the capsid and the tight grip on the DNA by the stalled ATPase may induce DNA bending, facilitating the headful cleavage.

While this model only describes the cleavage of one strand of the dsDNA substrate, producing a nicked DNA product, cleavage of the second strand may be achieved by a major reorientation of the terminase–DNA complex. Alternatively, cleavage of the second DNA strand can result from binding of a second large terminase, either recruited to the initiation complex, or present as a subunit within the pentameric motor (3) for the headful cleavage event. Further work will ascertain the validity of either of these models.

## ACCESSION NUMBERS

The genomic sequence for G20c has been deposited with the NCBI Genbank database, accession number KX987127. Structures of the G20c large terminase nuclease have been deposited with the Protein Data Bank, accession codes 5M1F (Apo), 5M1K (Mg^2+^), 5M1N (Mn^2+^), 5M1O (Co^2+^), 5M1P (Ca^2+^) and 5M1Q (Zn^2+^).

## Supplementary Material

Supplementary Data
