# Supplementary material for: Viral genome packaging terminase cleaves DNA using the canonical RuvC-like two-metal catalysis mechanism
Source: Nucleic Acids Res. 2017 Jan 18;45(6):3580–90. doi: 10.1093/nar/gkw1354 (PMC5389553; doi:10.1093/nar/gkw1354)
Supplement: Supplementary Data [file gkw1354_Supplementary_Data.zip › nar-03044-h-2016-File008.pdf]

Supplementary information for:

Viral genome packaging terminase cleaves DNA using the canonical RuvC-like two-metal catalysis mechanism

Rui-Gang Xu, Huw T. Jenkins, Maria Chechik, Elena V. Blagova, Anna Lopatina, Evgeny Klimuk, Leonid Minakhin, Konstantin Severinov, Sandra J. Greive, and Alfred A. Antson

Supplementary Figures

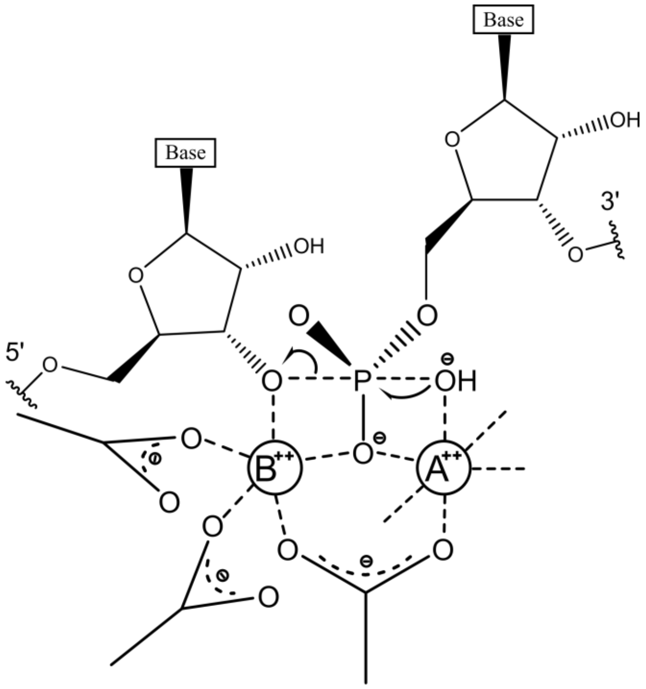

Supplementary Figure 1. RNase H catalytic mechanism. In the proposed mechanism (1), the transition state involves two metal ions that neutralize the developing negative charge.

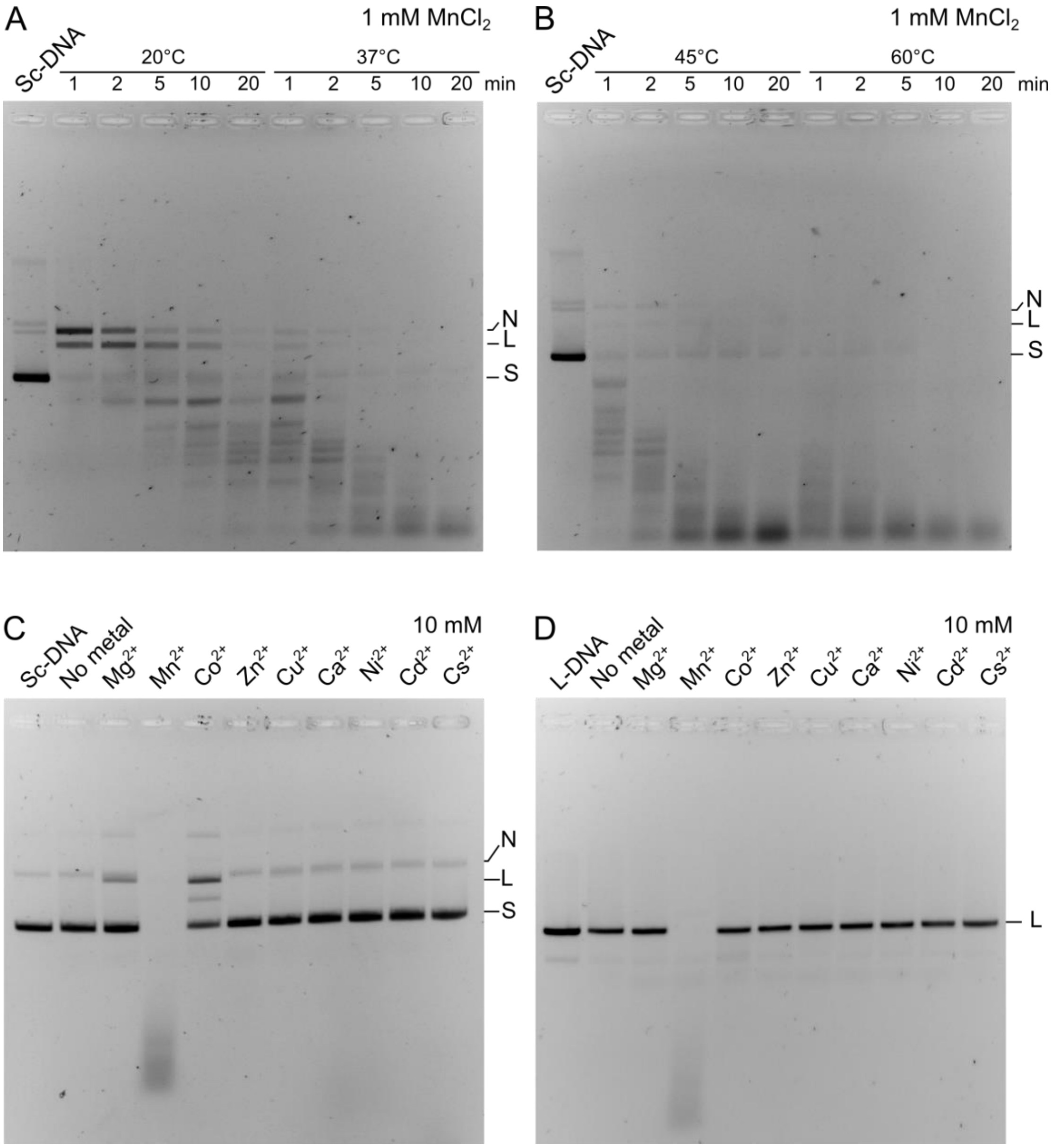

Supplementary Figure 2. Nuclease activity. Temperature dependence is shown at 1, 2, 5, 10 and 20 min at (A) 20 and 37°C or (B) 45 and 60°C. Effect of divalent metal ions on the nuclease activity is also shown using (C) supercoiled DNA or (D) linear DNA as substrate. DNA products were analysed using agarose gel electrophoresis. N, nicked; L, linear; S, supercoiled DNA

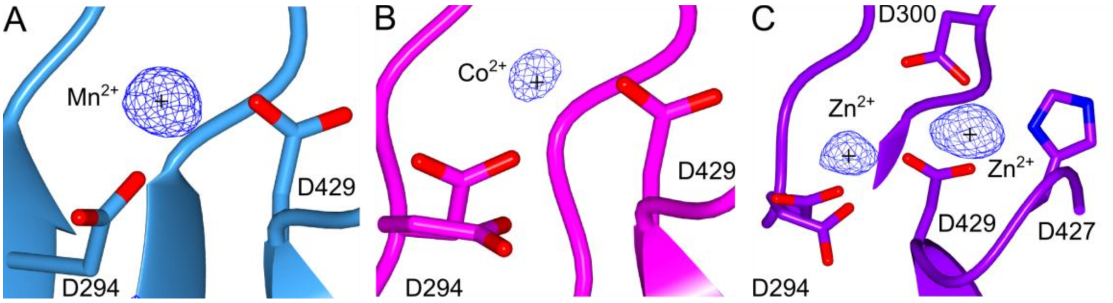

Supplementary Figure 3. Identification of metal binding sites. The anomalous difference electron density maps, contoured at 5 σ, are shown for (A) Mn^{2+}, (B) Co^{2+} and (C) Zn^{2+}.

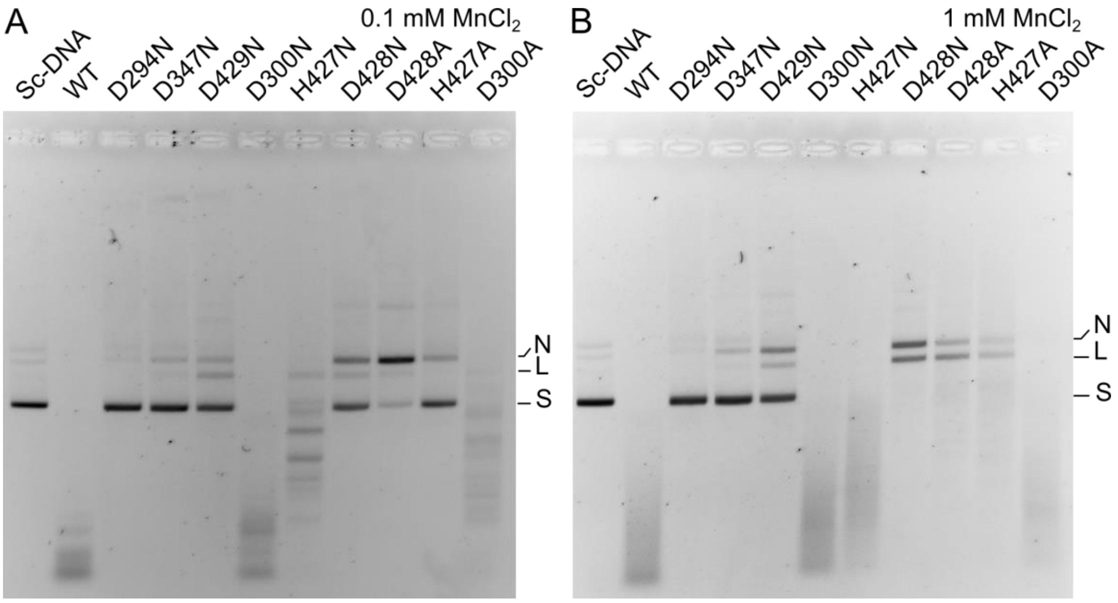

Supplementary Figure 4. Nuclease activity. Activity is shown for the wild type and active site mutant proteins in the presence of (A) 0.1mM $MnCl_2$ or (B) 1mM $MnCl_2$. DNA products were analysed using agarose gel electrophoresis. N, nicked; L, linear; S, supercoiled DNA

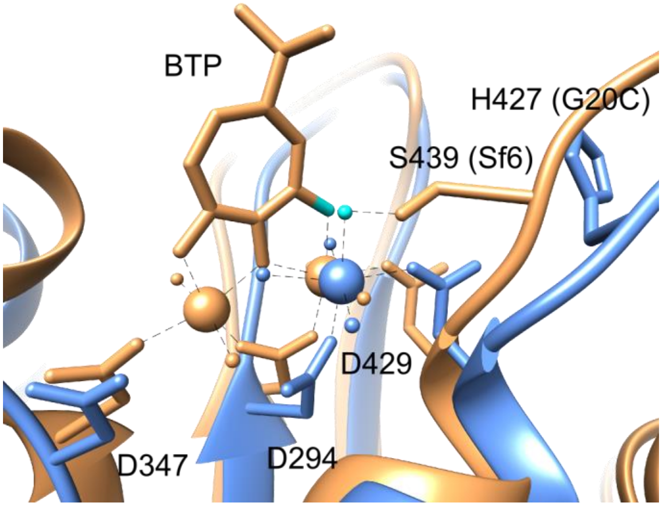

Supplementary Figure 5. Comparison of large terminases. Superposition of the Sf6 (brown) and G20c (blue) nucleases. The oxygen of β-thujaplicinol (BTP; cyan) occupies the same position as the nucleophile water. This oxygen forms a hydrogen bond with S439, equivalent to H427 in the G20c nuclease.

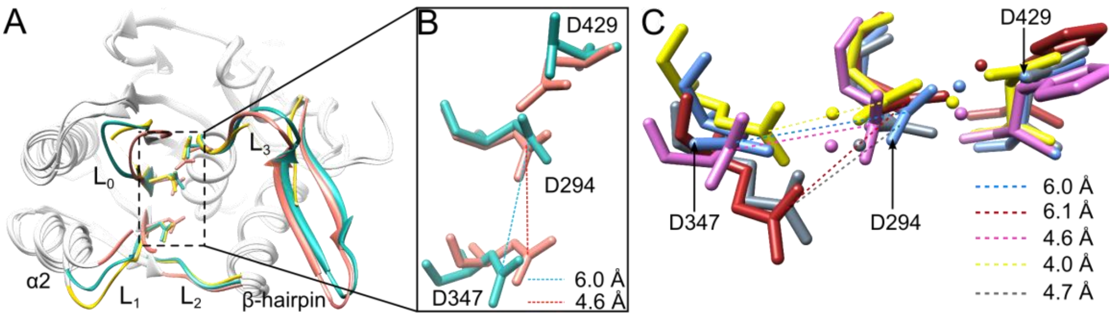

Supplementary Figure 6. Conformational flexibility in the active site. (A) Superposition of G20c structures with the most flexible regions highlighted (yellow, crystal form 1; salmon, crystal form 2; cyan, crystal form 3). (B) Close-up view of (A) with residues from crystal form 1 omitted. The Cγ-Cγ distances between D347 and D294 are indicated using dashed lines. (C) Superposition of the G20c (blue), SPP1 (purple) and HCMV (yellow) large terminase nucleases with RuvC resolvases from Canarypox virus (dark red) and *Lactococcus* phage bIL67 (grey). Distances between the two carboxyl groups coordinating metal B are shown.

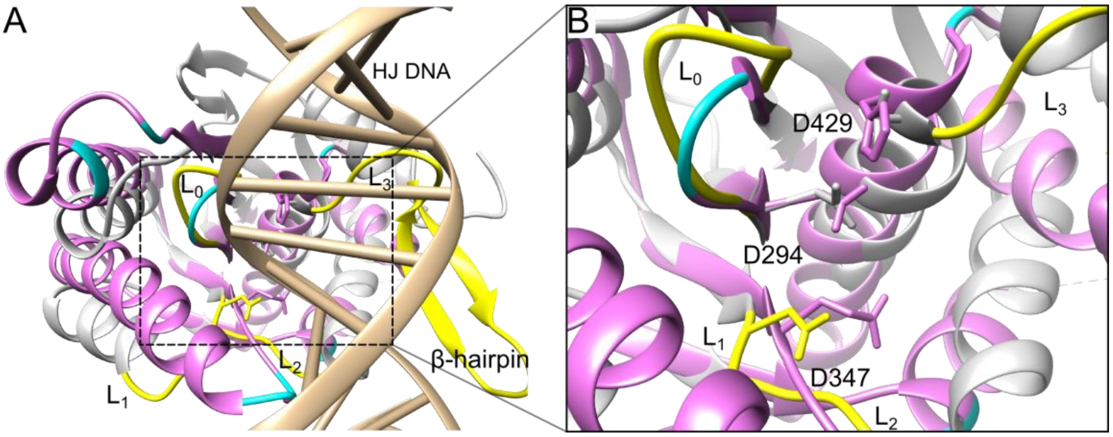

Supplementary Figure 7. Comparison of G20c large terminase and Tth-RuvC. (A) Superposition of Tth-RuvC resolvase (purple/cyan) and G20c nuclease (white/yellow). DNA is in beige. Regions of Tth-RuvC contacting DNA are in cyan. Loops of the G20c nuclease that were predicted to interact with DNA are in yellow. (B) A close-up view of the active site of the two proteins shown in (A).

Supplementary Table 1. Structure alignment of G20C large terminase nuclease with Bh-RNase H and Tth-RuvC (2).

	Q-score	P-score	Z-score	RMSD (Å)	N_{algn}	N_{sse}	Seq-%	N_{md}	Nres-Q
**RNase H**	0.117	-	1.86	4.1	93	4	0.043	4	135
**RuvC**	0.226	3.84	6.96	2.4	106	8	0.132	2	158

References

1. Nowotny, M. and Yang, W. (2006) Stepwise analyses of metal ions in RNase H catalysis from substrate destabilization to product release. *The EMBO Journal*, **25**, 1924-1933.

2. Krissinel, E. and Henrick, K. (2004) Secondary-structure matching (SSM), a new tool for fast protein structure alignment in three dimensions. *Acta Crystallographica Section D: Biological Crystallography*, **60**, 2256-2268.
